# Chronic ethanol feeding promotes azoxymethane and dextran sulfate sodium-induced colonic tumorigenesis potentially by enhancing mucosal inflammation

**DOI:** 10.1186/s12885-016-2180-x

**Published:** 2016-03-07

**Authors:** Pradeep K. Shukla, Kamaljit K. Chaudhry, Hina Mir, Ruchika Gangwar, Nikki Yadav, Bhargavi Manda, Avtar S. Meena, RadhaKrishna Rao

**Affiliations:** Department of Physiology, University of Tennessee Health Science Center, 894 Union Avenue, Nash 426, Memphis, TN 38163 USA

**Keywords:** Colon, Cancer, Alcohol, Cytokine, Chemokine, Inflammation, Tight junction

## Abstract

**Background:**

Alcohol consumption is one of the major risk factors for colorectal cancer. However, the mechanism involved in this effect of alcohol is unknown.

**Methods:**

We evaluated the effect of chronic ethanol feeding on azoxymethane and dextran sulfate sodium (AOM/DSS)-induced carcinogenesis in mouse colon. Inflammation in colonic mucosa was assessed at a precancerous stage by evaluating mucosal infiltration of neutrophils and macrophages, and analysis of cytokine and chemokine gene expression.

**Results:**

Chronic ethanol feeding significantly increased the number and size of polyps in colon of AOM/DSS treated mice. Confocal microscopic and immunoblot analyses showed a significant elevation of phospho-Smad, VEGF and HIF1α in the colonic mucosa. RT-PCR analysis at a precancerous stage indicated that ethanol significantly increases the expression of cytokines IL-1α, IL-6 and TNFα, and the chemokines CCL5/RANTES, CXCL9/MIG and CXCL10/IP-10 in the colonic mucosa of AOM/DSS treated mice. Confocal microscopy showed that ethanol feeding induces a dramatic elevation of myeloperoxidase, Gr1 and CD68-positive cells in the colonic mucosa of AOM/DSS-treated mice. Ethanol feeding enhanced AOM/DSS-induced suppression of tight junction protein expression and elevated cell proliferation marker, Ki-67 in the colonic epithelium.

**Conclusion:**

This study demonstrates that chronic ethanol feeding promotes colonic tumorigenesis potentially by enhancing inflammation and elevation of proinflammatory cytokines and chemokines.

## Background

Alcohol is identified by World Health Organization as one of the top ten risk factors for worldwide burden of disease [1]. A significant body of evidence indicates that chronic alcohol consumption increases the risk for cancer of many organs, including the respiratory tract, gastrointestinal tract, liver and breast [2–6]. International Agency for Research on Cancer concluded that alcohol is carcinogenic to humans at Group I category, the highest causality classification.

Colorectal cancer is the second common cause of death among adult cancer patients. Alcohol consumption is one of the major risk factors for colorectal cancer [7–9]. Although few animal studies failed to show a significant effect of ethanol on dimethylhydrazine (DMH)-induced colonic tumorigenesis [10–12], many studies showed that alcohol administration causes a significant increase in the incidence and tumor growth induced by DMH or methyl azoxymethanol (MAM) in rats [13–16]. Ethanol however was found to decrease the incidence of azoxymethane (AOM)-induced tumorigenesis in rats [17, 18]. Interestingly, tumorigenesis models in all these studies used the carcinogens to induce carcinogenesis without involving inflammation.

Although several mechanisms have been proposed [19], inflammation appears to be one of the major mechanisms associated with the development of colon cancer [20, 21]. An association between colorectal cancer and ulcerative colitis has been well established [22]. Histopathologic analysis indicates a role for inflammation in colon carcinogenesis in ulcerative colitis patients, which is confirmed in animal models of ulcerative colitis [21]. Ulcerative colitis patients complain of worsening gastrointestinal symptoms with alcohol consumption [23]. Alcohol consumption activates mast cells in response to oncogenic signals [24], suggesting that alcohol may have a significant impact on the intestinal mucosal inflammation.

In this study, we evaluated the effect of chronic ethanol feeding on AOM and dextran sulfate sodium (DSS)-induced tumorigenesis in mouse colon. Additionally, we analyzed the effect of ethanol feeding on colonic mucosal inflammation and expression of cytokines and chemokines at a precancerous stage.

## Methods

### Chemicals

DSS was purchased from Thermo Fisher Scientific (Tustin, CA) and AOM was purchased from Sigma-Aldrich (St. Louis, MO). Hoechst 33342 dye was purchased from Life technologies (Grand Island, NY). Precast, SDS-polyacrylamide gels were purchased from Invitrogen (Carlsbad, CA). All other chemicals were either from Sigma-Aldrich or Fisher Scientific.

### Antibodies

Anti-phospho Smad2 (pSmad; Cat#AB3849), anti-Ki-67 (Cat# AB9260), HRP-conjugated anti-rabbit IgG (Cat# 12–348), and anti-vascular endothelial growth factor (VEGF; Cat# 05–443) were purchased from Millipore (Billerica, MA). Anti-caspase-3 antibody (Cat# 9661) was from Cell Signaling Technology (Danvers, MA). Anti-CD68 antibody (Cat# 6A324) was purchased from Santa Cruz Biotechnology, Inc. (Santa Cruz, CA). Anti- hypoxia-inducible factor 1-alpha (HIF-1α; Cat#NB-100-449) was purchased from Novus Biologicals (Littleton, CO). Anti-occludin (Cat#331500) and anti-ZO-1 (Cat#617300) antibodies were purchased from Invitrogen. Anti-granulocyte receptor-1 (Gr1; Cat# MBS520313) was from MyBioSource (San Diego, CA). Horseradish peroxidase (HRP)-conjugated anti-mouse IgG (Cat# A4116), Cy3-conjugated anti-rabbit IgG (Cat# C2306) and anti-β-actin (Cat# A5441) antibodies were purchased from Sigma-Aldrich. Anti-myeloperoxidase (MPO) (Cat#DOM00001G), anti-claudin-3 (Cldn3; Cat# 341700), AlexaFlour 488-conjugated anti-mouse IgG (Cat# A11029), AlexaFluor 488-phalloidin (Cat# A12379), AlexaFluor 546-phalloidin (Cat# A22283), and anti-TNFα (Cat# AMC3012) antibodies were purchased from Thermo Fisher Scientific.

### Animals and diets

Female C57BL/6 mice (12–14 weeks, Harlan Laboratories, Houston, TX) were used for all experiments. All animal experiments were performed according to the protocol approved by the University of Tennessee Health Science Center-Institutional Animal Care and Use Committee. Animals were housed in institutional animal care facility with 12-h light and dark cycles and had free access to regular laboratory chow and water until the start of experiments. Lieber DeCarli diet (Dyet # 710260) was purchased from Dyets Inc. (Bethlehem, PA) and maltodextrin was from Bioserv (Flemington, NJ).

### Colonic tumorigenesis

Mice received one time injection of AOM (10 mg/kg BW) at the beginning of experiment. Five days after AOM injection, recurrent colitis was induced by administering DSS (3 % *w*/*v*) in drinking water; three 5-day courses with 15-day intervals. Tumorigenesis was observed at 30 days after the third DSS cycle. During the 15-day intervals and during the 30 days after 3rd DSS cycle, animals were fed Lieber-DeCarli liquid diet with or without 4 % ethanol (EtOH); non-EtOH group were pair fed isocaloric diet (adjusted with maltodextrin). For tumorigenesis studies (80 days total) 7 and 9 mice were used for AOM + DSS and AOM + DSS + EtOH, respectively. One mouse in AOM + DSS group and 2 mice in AOM + DSS + EtOH group died before the end of experiment. For precancerous studies (44 days period), 7 mice each were used for AOM + DSS and AOM + DSS + EtOH groups and no mice died in these experiments. Again 4 mice were used for control groups in all experiments. Colons were evaluated for polyp number and size. Cryosections were examined for carcinogenesis markers, pSmad, VEGF and HIF-1α.

### Quantitative RT-PCR

Total RNA (1.5 μg) was used for generation of cDNAs using the ThermoScript RT-PCR system for first strand synthesis (Invitrogen). Quantitative PCR (qPCR) reactions were performed using cDNA mix (cDNA corresponding to 35 ng RNA) with 300 nmoles of primers in a final volume of 25 μl of 2× concentrated RT2 Real-Time SYBR Green/ROX master mix (Qiagen) in an Applied Biosystems 7300 Real-Time PCR instrument (Norwalk, CT, USA). The cycle parameters were: 50 °C for 2 min, one denaturation step at 95 °C for 10 min and 40 cycles of denaturation at 95 °C for 10s followed by annealing and elongation at 60 °C. Relative gene expression of each transcript was normalized to GAPDH using the ΔΔCt method. Sequences of primers used for qPCR are provided in Table 1.

**Table 1:** Sequence of primers used for quantitative real-time PCR

Gene	5′-3′ Sequence
*TNF*-*α*	Forward: CCCTCACACTCAGATCATCTTCT
	Reverse: GCTACGACGTGGGCTACAG
*IL*-*1α*	Forward: CGAAGACTACAGTTCTGCCATT
	Reverse: GACGTTTCAGAGGTTCTCAGAG
*IL*-*6*	Forward: TAGTCCTTCCTACCCCAATTTCC
	Reverse: TTGGTCCTTAGCCACTCCTTC
*CXCL9*/*MIG*	Forward: GGAGTTCGAGGAACCCTAGTG
	Reverse: GGGATTTGTAGTGGATCGTGC
*CXCL10*/*IP*-*10*	Forward: CCAAGTGCTGCCGTCATTTTC
	Reverse: GGCTCGCAGGGATGATTTCAA
*CCL5*/*RANTES*	Forward: GCTGCTTTGCCTACCTCTCC
	Reverse: TCGAGTGACAAACACGACTGC
*IL*-*1β*	Forward: GCAACTGTTCCTGAACTCAACT
	Reverse: ATCTTTTGGGGTCCGTCAACT
*Ocln*	Forward: TTGAAAGTCCACCTCCTTACAGA
	Reverse: CCGGATAAAAAGAGTACGCTGG
ZO-1 (*TJP1*)	Forward: GCCGCTAAGAGCACAGCAA
	Reverse: TCCCCACTCTGAAAATGAGGA
*Cldn3*	Forward: ACCAACTGCGTACAAGACGAG
	Reverse: CAGAGCCGCCAACAGGAAA
*GAPDH*	Forward: CTGCACCACCAACTGCTTAG
	Reverse: GGGCCATCCACAGTCTTCT

### Immunofluorescence microscopy

Colons were examined for inflammatory markers MPO (neutrophil), Gr1 (neutrophil) and CD68 (macrophage) by confocal microscopy at a precancerous stage and tumorigenesis markers were examined when polyps were fully developed. Cryo-sections of distal colon (10 μm thickness) were fixed in acetone:methanol mixture (1:1) at 20 °C for 2 min and rehydrated in phosphate buffered saline (PBS). Sections were permeabilized with 0.2 % Triton X-100 in PBS for 15 min and blocked in 4 % non-fat milk in TBST (20 mM Tris, pH 7.2 and 150 mM NaCl). It was then incubated for 1 h with primary antibodies (rabbit polyclonal anti-pSmad, VEGF, Ki67, ZO1, Cldn3 and HIF-1α or mouse monoclonal anti-occludin; all at 1:100 dilution) followed by incubation with secondary antibodies (Cy3-conjugated anti-rabbit IgG antibodies at 1:100 dilution; Molecular Probes, Eugene, OR) and co-stained with AlexaFlour 488-conjugated phalloidin and Hoechst 33342 for 1 h. The fluorescence was examined by using a confocal microscope (Zeiss 710) and images from x-y sections (1 μm) were collected using Zen software. Images were stacked using the Image J software (NIH, Bethesda, MD) and processed by Adobe Photoshop (Adobe Systems Inc., San Jose, CA).

### Preparation of mucosal extract

Mucosal scrapping from distal colon was incubated on ice for 15 min with lysis buffer-CS (Tris buffer containing 1 % Triton-X100, 2 μg/ml leupeptin, 10 μg/ml aprotinin, 10 μg/ml bestatin, 10 μg/ml pepstatin-A, 10 μl/ml of protease inhibitor cocktail, 1 mM sodium orthovanadate and 1 mM phenylmethyl sulfonylfluoride. Mucosal lysates were centrifuged at 15,600 × g for 4 min at 4 °C to collect total fraction. Protein content was measured by BCA method (Pierce Biotechnology, Rockford, IL). Total fraction was mixed with equal volume of Laemmli’s sample buffer (2× concentrated), heated at 100 °C for 5 min, and 40 μg proteins from each sample was used for immunoblot analysis.

### Immunoblot analysis

Total mucosal fractions were separated by SDS-polyacrylamide gel (7 %) electrophoresis and transferred to PVDF membranes. Membranes were immunoblotted for different proteins using specific antibodies for anti-occludin (1:250 dilution), pSmad (1:1000 dilution) and VEGF with β-actin (1:5000 dilution) as house keeping protein in combination with HRP-conjugated anti-mouse IgG or anti-rabbit IgG secondary antibodies (1:2000 dilution). The blots were developed using ECL chemiluminescence method (Pierce, Rockford, IL) and quantitated by densitometry using Image J software. The density for each band was normalized to density of corresponding actin band.

### Statistical analyses

All data are expressed as Mean ± SEM. The differences among multiple groups were first analyzed by ANOVA (Prism 6.0). When a statistical significance was detected, Tukey’s *t* test was used to determine the statistical significance between multiple testing groups and the corresponding control. Statistical significance was established at 95 %.

## Results

### Ethanol feeding promotes AOM/DSS-induced colonic tumorigenesis

**Fig. 1:**
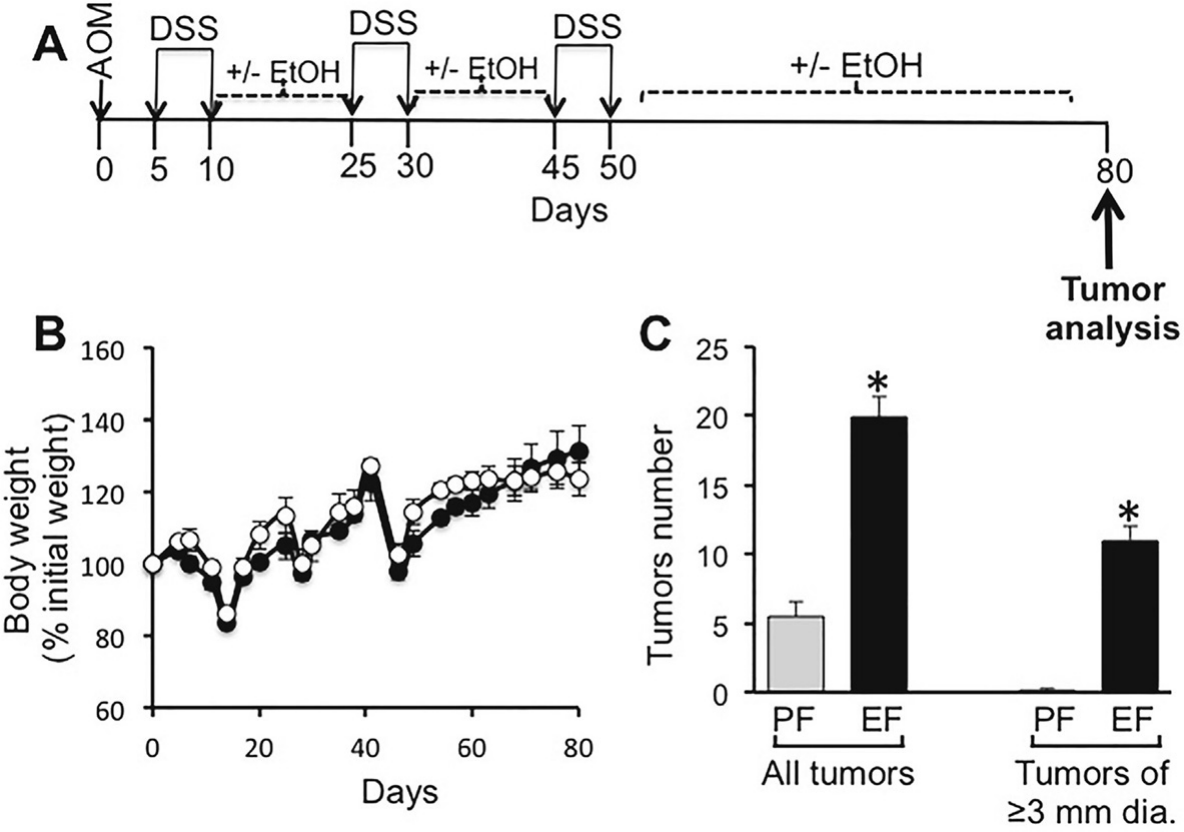
Ethanol feeding increases AOM/DSS-induced tumorigenesis in mouse colon. **a** The scheme shows one time administration of azoxymethane (AOM) followed by three course of DSS (dextran sodium sulfate)-induced colitis and separated by 15-day recovery periods. Colon was examined 30 days after the third course of DSS colitis. During all recovery periods, animals were fed liquid diet with or without 4 % ethanol (EtOH) or isocaloric maltodextrin. **b** Body weights were recorded twice a week. Values represent the mean of 7–9 samples per group. **c** The number of tumors per colon was counted and the tumor size evaluated by measuring the diameter in AOM/DSS-treated mice with (EF) or without (PF) EtOH feeding. Values for total tumors per colon and tumors of 3 mm or greater diameter are mean ± SEM (*n* = 6 for PF and 7 for EF). *Asterisks* indicate the values that are significantly different (*p* < 0.05) from corresponding values for PF group

**Fig. 2:**
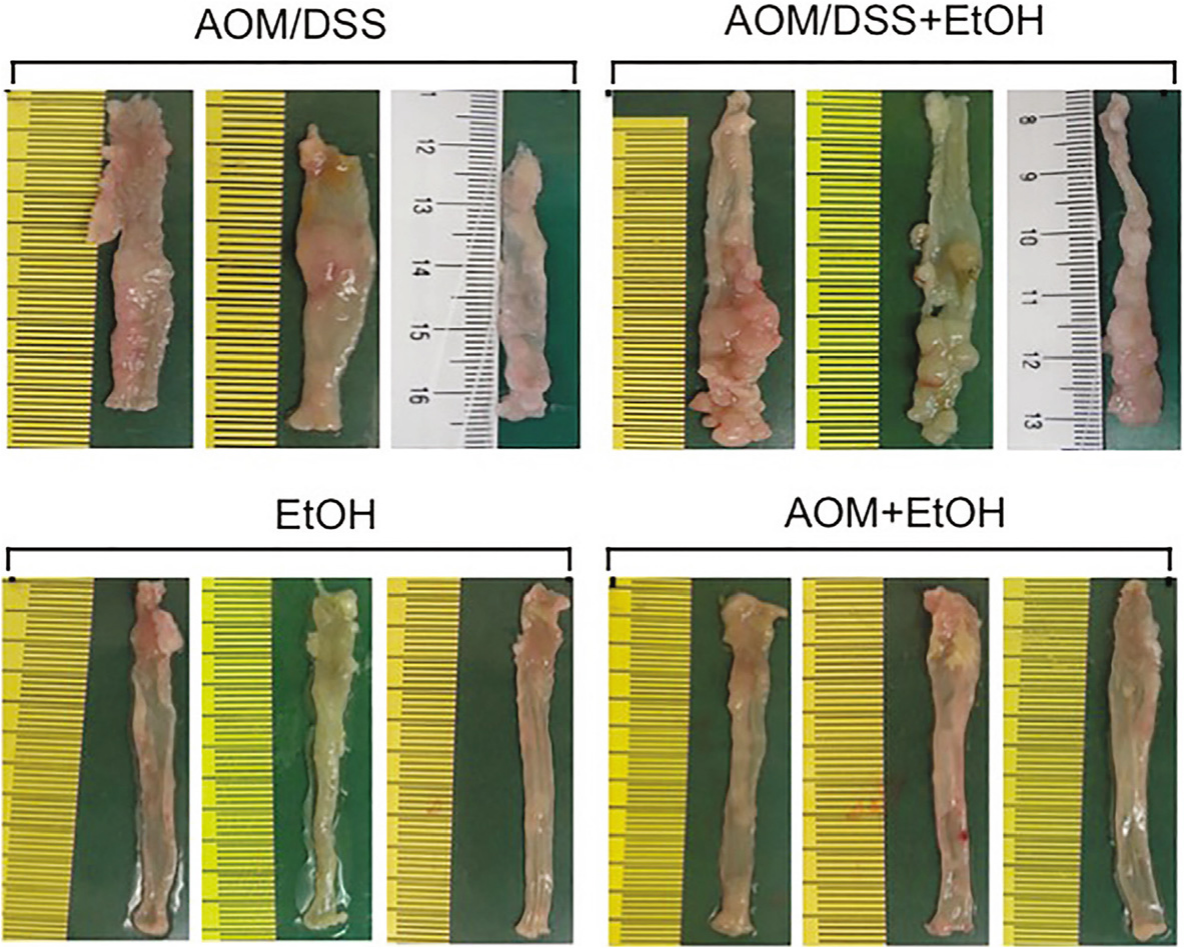
Ethanol feeding increases AOM/DSS-induced tumorigenesis in mouse colon. Photographs of colons from 3 animals per group are presented

Ethanol was fed during the post colitis recovery period of AOM/DSS-induced colonic tumorigenesis, which is likely to have an influence on post colitis healing (Fig. 1a). Ethanol feeding did not cause a significant alteration of body weight during the AOM/DSS-induced tumorigenesis (Fig. 1b). As shown before, AOM/DSS treatment resulted in the development of tumors in the distal colon, which was significantly elevated by ethanol feeding. Total number of tumors was elevated nearly 4-fold by ethanol feeding (Fig. 1c). Interestingly, in the absence of ethanol, diameter of all tumors was less than 3 mm. On the other hand, diameter of more than half of the tumors in ethanol fed mice was 3 mm or greater (Fig. 1c). Representative photographs of colons from mice in different groups are presented in Fig. 2.

### Ethanol feeding enhances tumorigenic markers in the colon

**Fig. 3:**
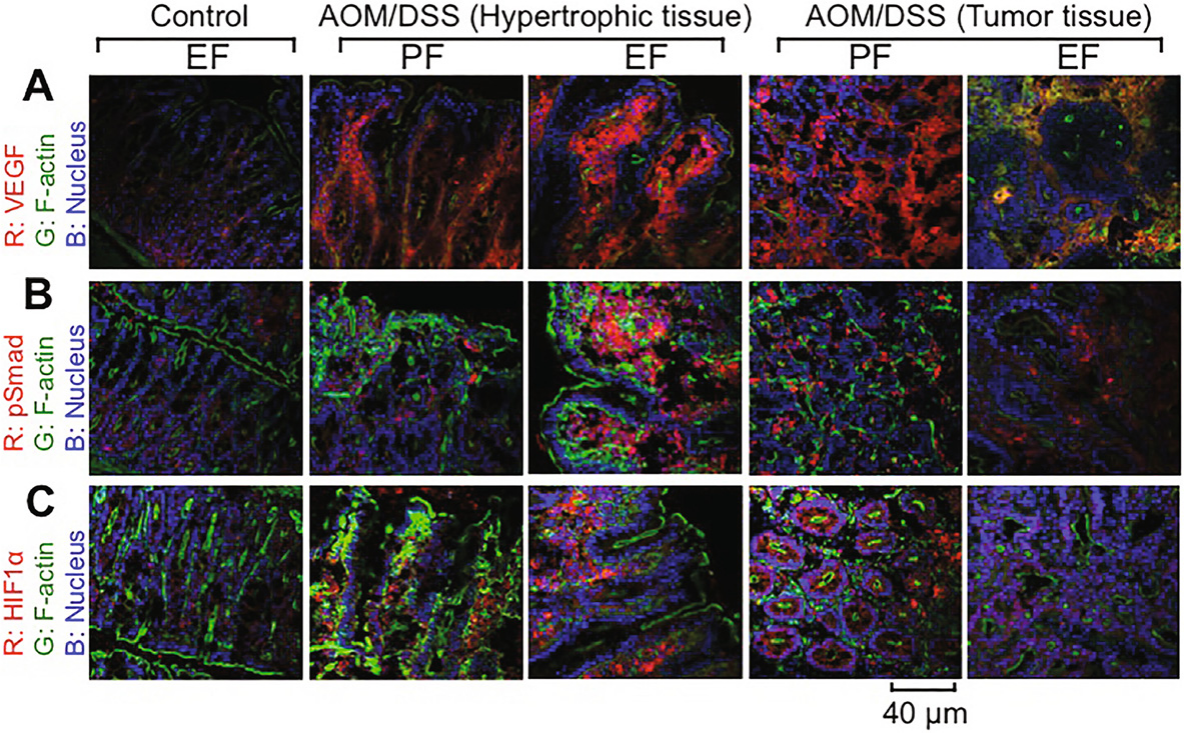
Chronic ethanol feeding enhances tumorigenesis markers during AOM/DSS-induced tumorigenesis. AOM/DSS-induced colonic tumorigenesis was induced with (EF) or without (PF) 4 % ethanol feeding as described in Methods section. Cryosections of distal colon were stained for VEGF (**a**) pSmad (**b**), or HIF1α (**c**), and co-stained for F-actin and nucleus. Images presented are representative of *n* of 5 for each group

We analyzed the levels of the tumor markers, VEGF, pSmad and HIF1α, by immunofluorescence microscopic method. Images from the tumor tissue as well as the non-tumor hypertrophic colonic mucosa in the same colon were separately examined. Low levels of VEGF stain was present in the colon of ethanol fed-AOM/DSS treated mice, but relatively high levels of VEGF stain was present in the non tumor hypertrophic colonic mucosa of AOM/DSS treated mice with or without ethanol feeding (Fig. 3a). In the hypertrophic colonic mucosa, VEGF was localized predominantly in the lamina propria region. High level of VEGF was also detected in the tumor tissue, where it was distributed predominantly in the sub epithelial connective tissue regions. The stain for pSmad was very low in hypertrophic colonic mucosa in the ethanol fed or AOM/DSS treated mice, but the stain was dramatically higher in colon of ethanol fed-AOM/DSS treated mice (Fig. 3b). Stain for pSmad was also localized predominantly in the lamina propria. Higher levels of stain for pSmad were detected in the tumor tissues in AOM/DSS treated mice with or without ethanol feeding. HIF1α level was low in the colon of ethanol fed control mice, and AOM/DSS treatment had a very little effect on HIF1α level in the hypertrophic colon (Fig. 3c). Ethanol feeding dramatically elevated HIF1α stain in the hypertrophic colon of AOM/DSS treated mice, which once again was localized predominantly in lamina propria. Higher level of stain for HIF1α was present in the tumor tissues, but ethanol appeared to reduce this stain.

**Fig. 4:**
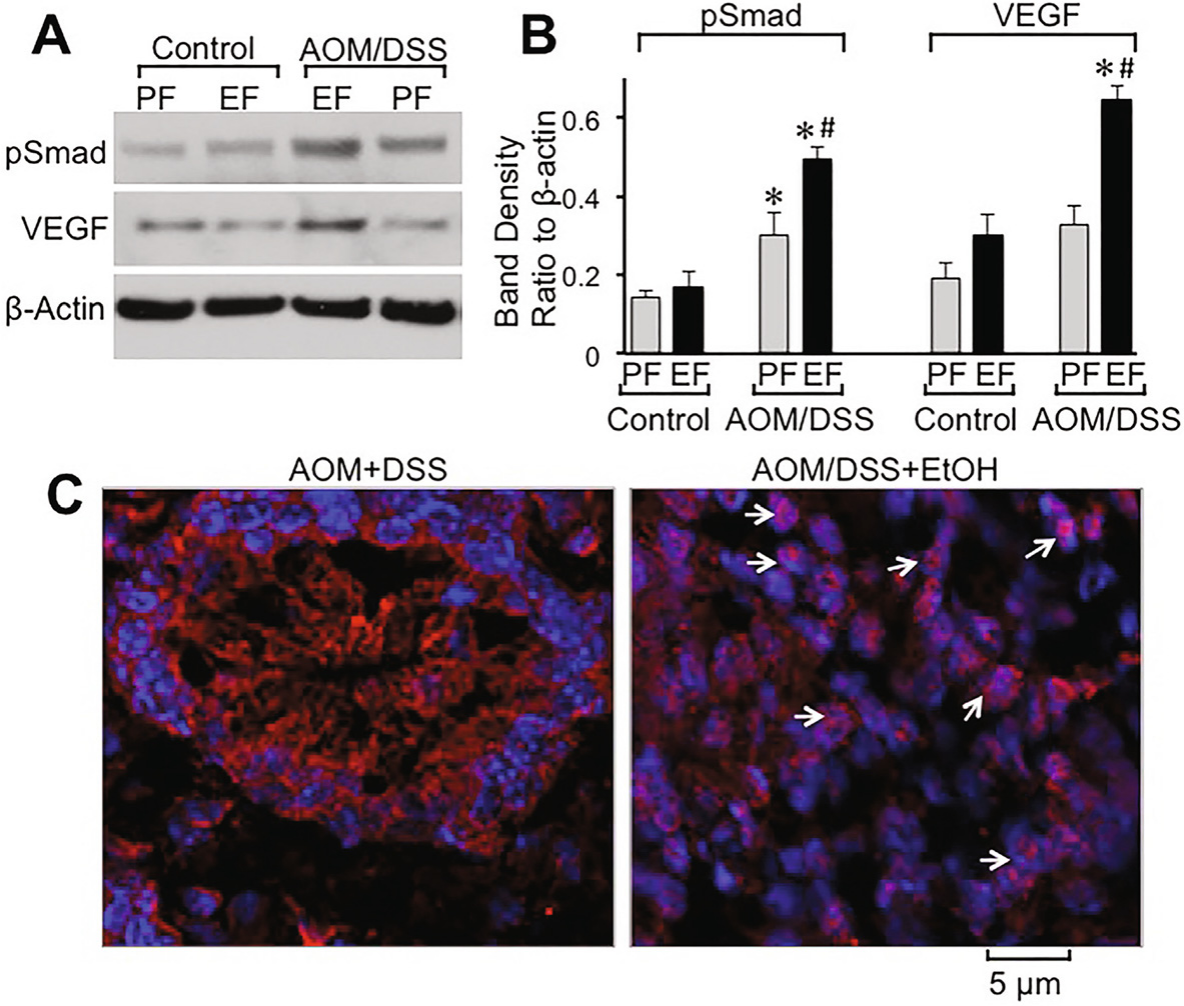
Ethanol elevates tumorigenic markers and nuclear localization of HIF1α in colonic crypts during AOM/DSS-induced tumorigenesis: AOM/DSS-induced colonic tumorigenesis was induced with or without 4 % ethanol feeding as described in Methods section. **a & b** Colonic mucosal extracts were immunoblotted for pSmad and VEGF (**a**), and the band density evaluated using Image J software (**b**). Values are mean ± SE (*n* = 3). *Asterisks* indicate the values that are significantly different (*p* < 0.05) from corresponding values for AOM/DSS group. **c** Cryosections of colon were stained for HIF1α (*red*) and nucleus (*blue*). Sections of the images from Fig. 2c are enlarged to show the detailed localization of HIF1α in the nucleus. While *arrows* indicate nuclear co-localization of HIF1α

Immunoblot analysis of hypertrophic colonic mucosal extracts showed low levels of pSmad in pair fed and ethanol fed controls, which was significantly elevated in AOM/DSS treated mice (Fig. 4a). Ethanol feeding further elevated pSmad in the colon of AOM/DSS treated mice (Fig. 4a and b). Similarly, significant level of VEGF was present in the colon of ethanol control mice. The level of VEGF was unaffected by AOM/DSS treatment, but ethanol feeding significantly elevated VEGF stain in the colon of AOM/DSS treated mice. Enlarging images for HIF1α in the crypt region of hypertrophic colon from AOM/DSS and ethanol fed-AOM/DSS treated mice show that ethanol feeding enhanced nuclear translocation of HIF1α (Fig. 4c).

### Ethanol causes sustained inflammation in the colon of AOM/DSS-treated mice

**Fig. 5:**
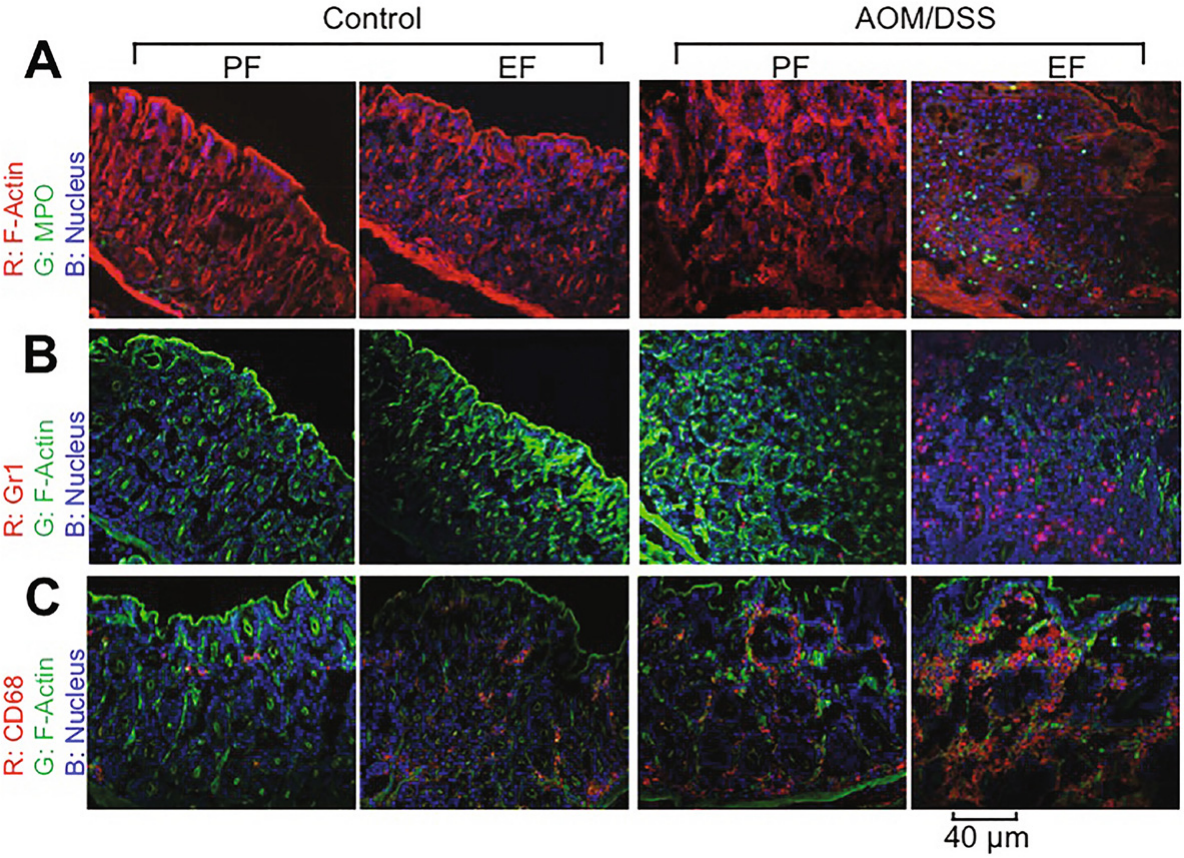
Ethanol elevates inflammatory cells in colonic mucosa of AOM/DSS-treated mice: AOM/DSS-induced colonic tumorigenesis was induced with (EF) or without (PF) 4 % ethanol feeding as described in Methods section. At the pre-cancerous stage (Day 44; 2 week after second course od DSS colitis) cryosections of distal colon were stained for myeloperoxidase (MPO) (**a**), Gr1 (**b**) and CD68 (**c**) and co-stained for F-actin and nucleus. Images presented are representative of n of 5 for each group

At 2 weeks after the second course of DSS colitis (Fig. 1a), we examined colonic mucosa for inflammation by staining colonic sections for MPO, Gr1 (neutrophil marker) or CD68 (macrophage marker) by immunofluorescence staining method. Colon from ethanol control mice and AOM/DSS treated mice showed no stain for MPO-positive cells (Fig. 5a). But, there were numerous MPO-positive cells present in the colonic mucosa of ethanol fed-AOM/DSS treated mice. Similarly, Gr1-positive cells were not detectable in the colon of ethanol or AOM/DSS treated mice (Fig. 5b), but numerous Gr1-positive cells were present in the colonic mucosa of ethanol fed-AOM/DSS treated mice. CD68-positive cells were detected in the colonic mucosa of ethanol fed and AOM/DSS treated mice (Fig. 5c). CD68-positive cells appear to be relatively high in the colon of ethanol fed-AOM/DSS treated mice.

### Ethanol feeding elevates cytokine and chemokine gene expression in colonic mucosa of AOM/DSS treated mice

**Fig. 6:**
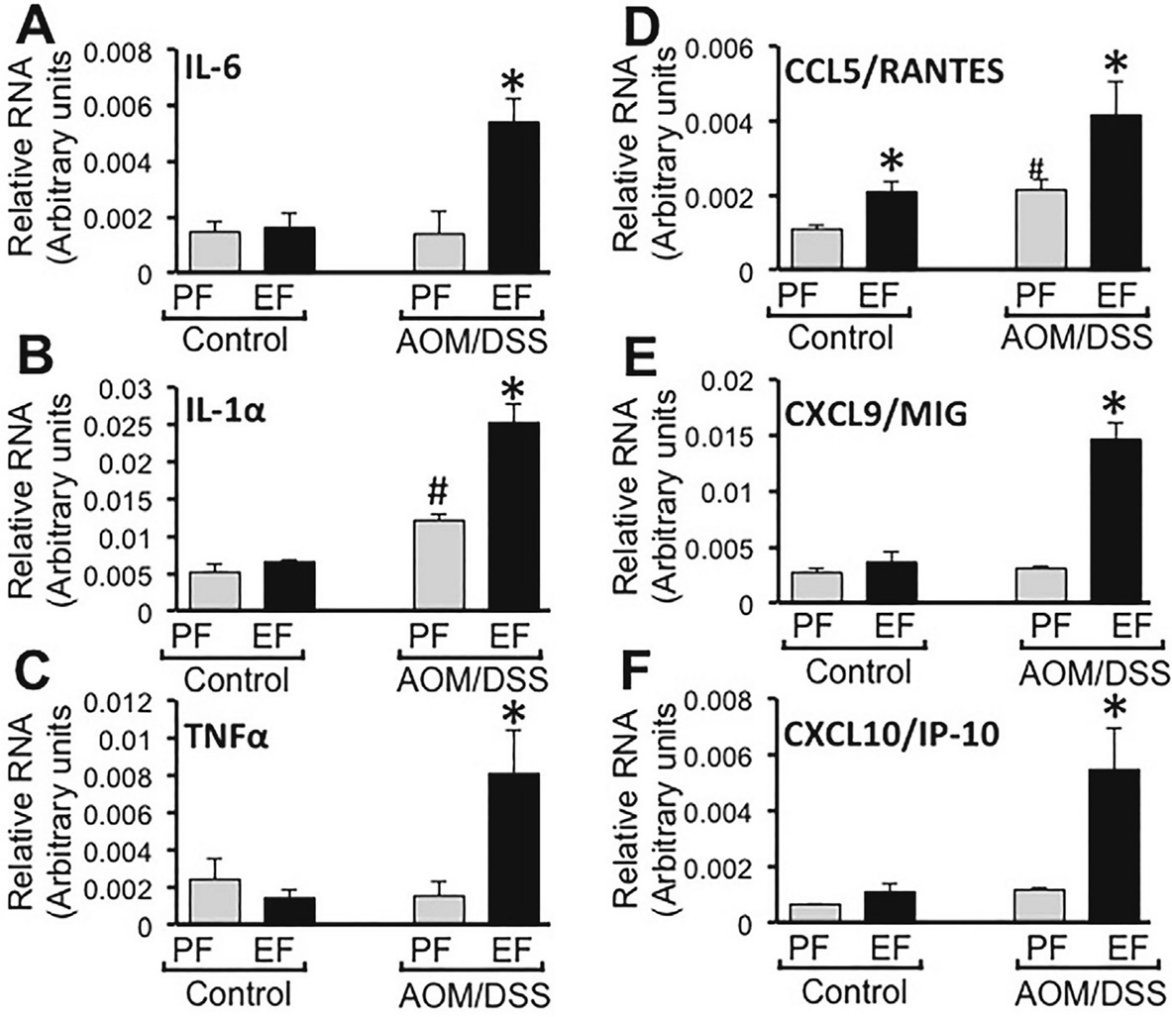
Chronic ethanol feeding promotes cytokine and chemokine gene expression in AOM/DSS-induced colonic tumorigenesis. AOM/DSS-induced colonic tumorigenesis was induced with (EF) or without (PF) 4 % ethanol feeding as described in Methods section. At the pre-cancerous stage (Day 44; 2 week after second course od DSS colitis), qRT-PCR analysis performed in RNA from distal colonic mucosa for IL-6 (**a**), IL-1α (**b**), TNFα (**c**), CCL5/RANTES (**d**), CXCL9/MIG (**e**) and CXCL10/IP-10 (**f**). Values are mean ± SE (*n* = 7). *Asterisks* indicate the values that are significantly (*p* < 0.05) different from corresponding PF values. *Hash tag* symbol indicates the value that is significantly (*p* < 0.05) different from corresponding “Control” value

To determine the effect of ethanol feeding on post colitis recovery, we measured mRNA for cytokines and chemokines at a precancerous stage, that is 2 weeks after the second course of DSS colitis (Fig. 1a). The levels of mRNA for IL-6 in colonic mucosa of ethanol control and AOM/DSS treated mice were not different from those in control mice (Fig. 6a). IL-6 mRNA level was nearly 4-fold higher in colonic mucosa of ethanol fed-AOM/DSS treated mice. IL-1α mRNA levels were similar in the colon of pair fed control and ethanol fed mice (Fig. 6b). But, it was about 2-fold higher in the colon of AOM/DSS treated mice and nearly 5-fold higher in the colon of ethanol fed-AOM/DSS treated mice. Similar to IL-6 mRNA, the levels of mRNA for TNFα in the colon of pair fed control, ethanol fed and AOM/DSS treated mice were not significantly different from each other (Fig. 6c). But, it was 4-5-fold higher in the colon of ethanol fed-AOM/DSS treated mice (Fig. 6c). The levels of mRNA for IL-1β were not different in different groups (data not presented).

CCL5/RANTES mRNA level was significantly higher in the colon of ethanol fed mice compared to that in pair fed mice (Fig. 6d). But, it was nearly 4-fold higher in the colon of ethanol fed-AOM/DSS treated mice. The levels of mRNA for CXCL9/MIG and CXCL10/IP-10 were not significantly different in the colons of pair fed control, ethanol control and AOM/DSS treated mice (Fig. 6e and f). But, they were 5-8-fold higher in the colon of ethanol fed-AOM/DSS treated mice.

**Fig. 7:**
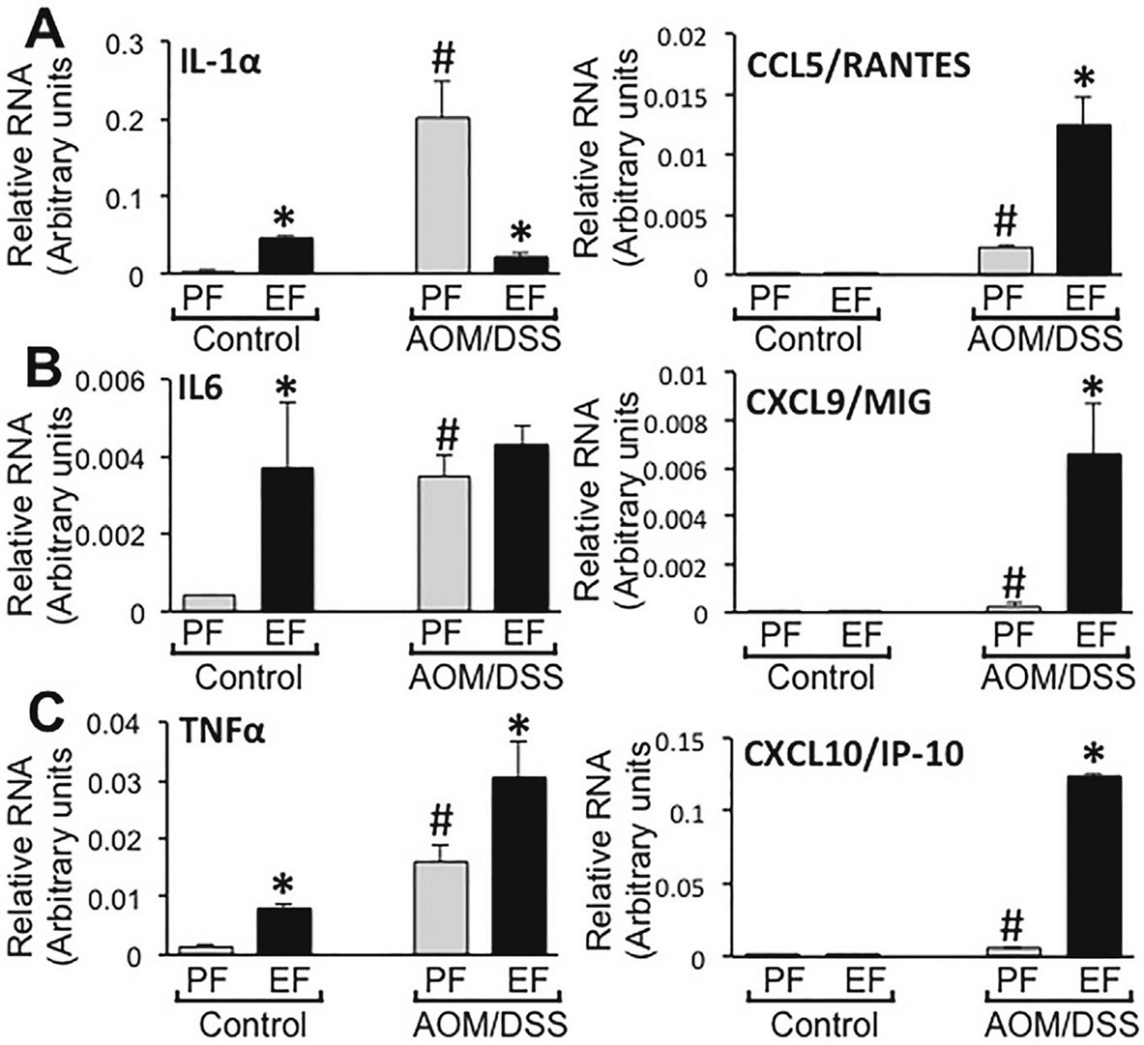
Chronic ethanol feeding modulates cytokines gene expression in the ileum of AOM/DSS treated mice. AOM/DSS-induced colonic tumorigenesis was induced with (EF) or without (PF) 4 % ethanol feeding as described in Methods section. At the pre-cancerous stage (Day 44; 2 week after second course of DSS colitis), qRT-PCR analysis performed in RNA from ileal mucosa for IL-6 (**a** left), IL-1α (**b** left), TNFα (**c** left), CCL5/RANTES (**a** right), CXCL9/MIG (**b** right) and CXCL10/IP-10 (**c** right). Values are mean ± SE (*n* = 4). *Asterisks* indicate the values that are significantly (*p* < 0.05) different from corresponding PF values. *Hash tag* symbols indicate the values that are significantly (*p* < 0.05) different from corresponding “Control” values

Cytokines mRNA levels were also measured in the ileum. Unlike in colon, the mRNA for IL-1α (Fig. 7a), IL-6 (Fig. 7b) and TNFα (Fig. 7c) in ileum were significantly higher in ethanol fed mice compared to that in pair fed control mice. IL-1α, IL-6 and TNFα mRNA levels were significantly higher in the ileum of AOM/DSS-treated mice. In AOM/DSS treated mice, ethanol feeding reduced the mRNA for IL-1α and increased TNFα mRNA, but had no effect on IL-6 mRNA levels. CCL5/RANTES (Fig. 7d), CXCL9/MIG (Fig. 7e) and CXCL10/IP-10 (Fig. 7f) mRNA levels in the ileum were slightly, but significantly higher in AOM/DSS-treated mice, but they were several folds higher in the ethanol fed-AOM/DSS treated mice.

### Ethanol promotes AOM/DSS-induced suppression of tight junction protein gene expression and elevation of cell proliferation marker

**Fig. 8:**
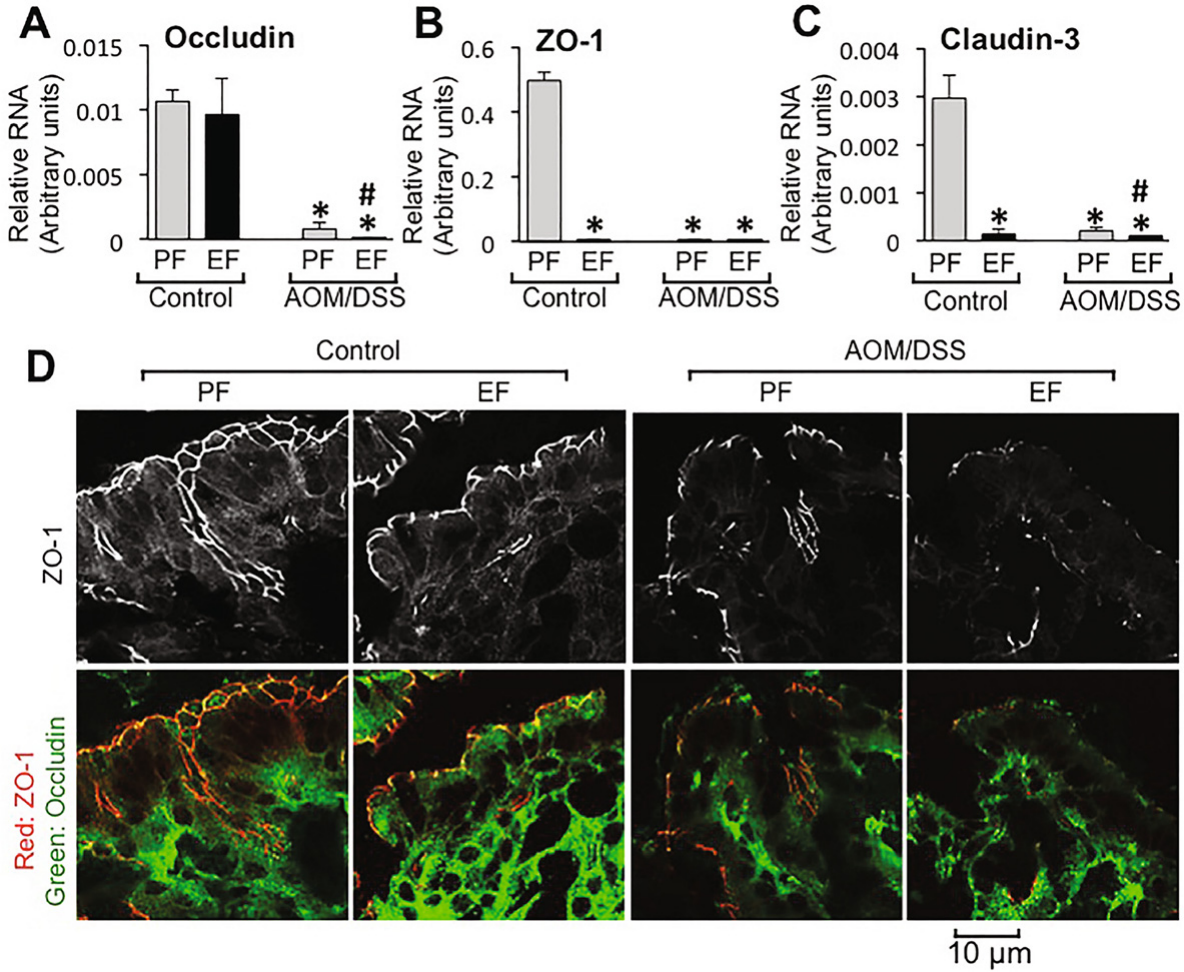
Chronic ethanol feeding modulates gene expression of tight junction proteins in the colon of AOM/DSS treated mice. AOM/DSS-induced colonic tumorigenesis was induced with (EF) or without (PF) 4 % ethanol feeding as described in Methods section. **a**-**c** At the pre-cancerous stage (Day 44; 2 week after second course of DSS colitis), qRT-PCR analysis performed in RNA from distal colonic mucosa for occludin (**a**), ZO-1 (**b**) and claudin-3 (**c**). Values are mean ± SEM (*n* = 4). *Asterisks* indicate the values that are significantly (*p* < 0.05) different from corresponding PF values. *Hash tag* symbols indicate the values that are significantly (*p* < 0.05) different from corresponding “Control” values. **d** Cryosections of colon were stained for occludin (*green*) and ZO-1 (*red*)

Occludin (Fig. 8a), ZO-1 (Fig. 8b) and Cldn3 (Fig. 8c) mRNA levels were dramatically low in AOM/DSS-treated mouse colon, and ethanol feeding further suppressed the mRNA for these proteins. Ethanol by itself did not affect occludin mRNA, but almost completely depleted ZO-1 and Cldn3 mRNA. Immunofluorescence staining showed only a slight effect on occludin and ZO-1 protein levels (Fig. 8d). AOM/DSS treatment caused a more severe depletion of occludin and ZO-1, which was further suppressed by ethanol feeding.

**Fig. 9:**
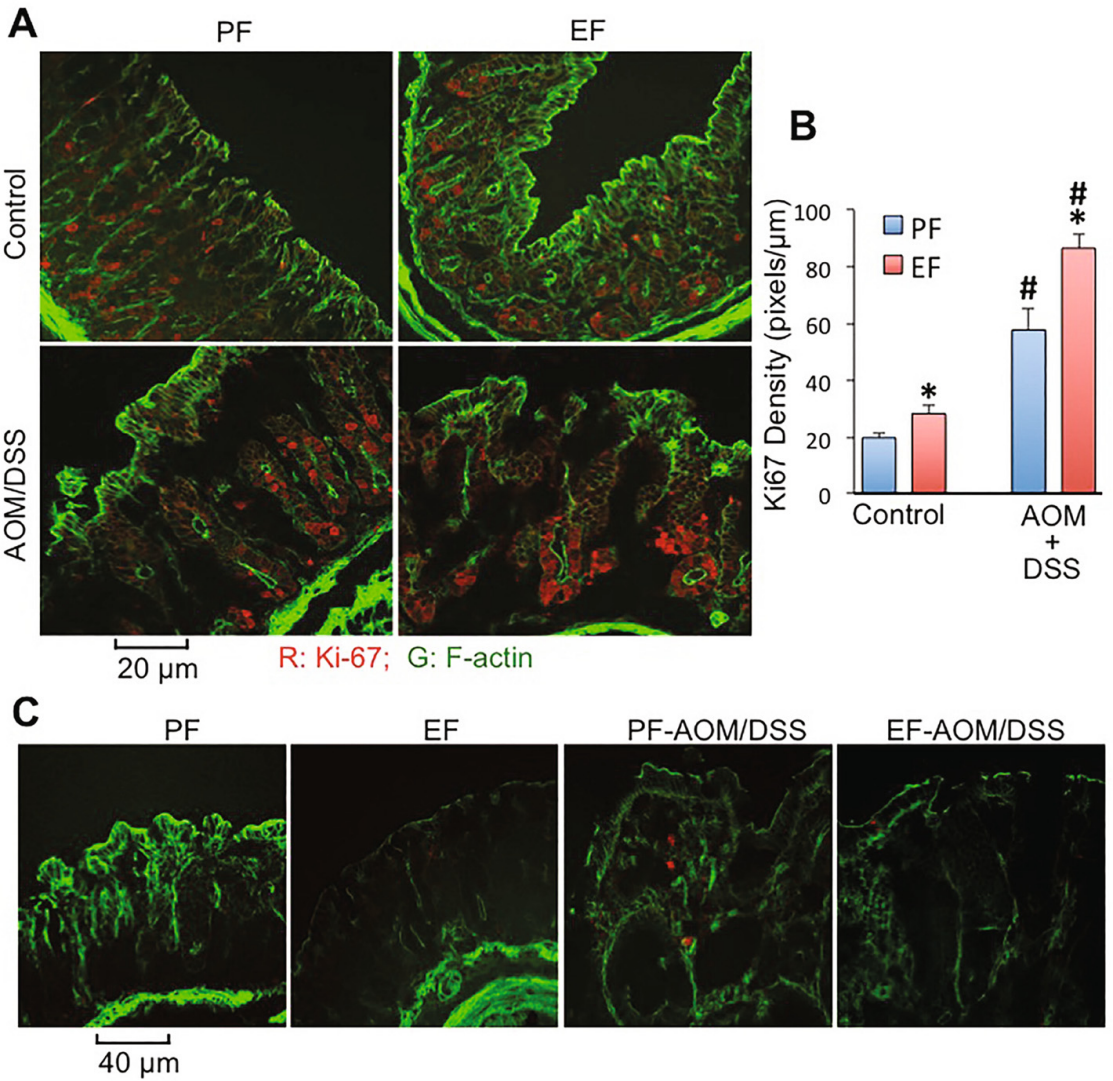
Chronic ethanol feeding promotes AOM/DSS-induced elevation of Ki67 in mouse colon. AOM/DSS-induced colonic tumorigenesis was induced with (EF) or without (PF) 4 % ethanol feeding as described in Methods section. At the pre-cancerous stage (Day 44; 2 week after second course of DSS colitis), colonic sections were stained for Ki67 (*red*) and F-actin (*green*) (**a**). Red fluorescence density in Ki67-positive cells was measured by densitometry (**b**). Fluorescence values are mean ± SEM (*n* = 5; each n is average of 8–10 cells in each section). *Asterisks* indicate the values that are significantly (*p* < 0.05) different from corresponding PF values. *Hash tag* symbols indicate the values that are significantly (*p* < 0.05) different from corresponding “Control” values. Colonic sections were also co-stained for F-actin (*green*) and activated caspase-3 (*red*) (**c**)

The number of Ki-67-positive cells in the colon was not different in ethanol and pair fed mice with or without AOM/DSS treatment (Fig. 9a). However, the fluorescence intensity in positive cells was significantly high in the colon of AOM/DSS treated mice, which was further elevated by ethanol feeding (Fig. 9b). Ethanol, in the absence of AOM/DSS, also showed a slight by significant increase in Ki-67 levels. Staining for caspase-3 showed no positive cells in the colon of all groups, except for detection of few likely hematopoietic cells in the lamina propria of colon from AOM/DSS treated mice (Fig. 9c).

## Discussion

Alcohol consumption is one of the major risk factors for colorectal cancer [7–9]. Many experimental studies showed that ethanol feeding significantly increases the incidence of DMH or MAM-induced tumor growth in rat colon [13–16], and promotes tumor invasion in C57BL/6-*APC*^*Min*^ mice [24]. However, ethanol was found to decrease the incidence of AOM-induced tumorigenesis in rats [17, 18]. A significant body of evidence indicates that inflammation is an important second hit in the development of most types of cancer including colon cancer [25]. Therefore, in this study we used AOM/DSS-induced colon carcinogenesis model that combines the effects of a carcinogen and colitis to evaluate the effect of ethanol consumption on colon cancer. Results of this study explicitly provide robust evidence to ethanol-mediated promotion of AOM-induced tumorigenesis in mouse colon potentially by enhancing mucosal inflammation.

Our study shows that chronic ethanol feeding causes a dramatic increase in the number and size of tumors in AOM/DSS treated mice. AOM and DSS treatments have previously been shown to induce tumorigenesis in the distal colon [26–28]. The present study, for the first time, demonstrates that chronic ethanol feeding enhances AOM/DSS-induced tumorigenesis in mouse colon. Although previous studies have shown that ethanol increases the incidence of colonic tumorigenesis induced by different carcinogens, the ethanol effect on-induced tumorigenesis is not known. Previous studies showed that high dose of AOM administration induces colonic tumorigenesis in Fischer 344 rats with 50 % prevalence [17, 18], but ethanol feeding (11–33 % calories) resulted in a dose-dependent reduction of prevalence of tumorigenesis. This is in contrast to the results of our present study. The difference between our study and the previous studies is that we combined AOM with DSS-induced colitis. The prevalence of tumorigenesis in AOM/DSS model is greater than 90 %, supporting the view that inflammation plays a key role in the promotion of colon cancer. This is consistent with the clinical observation showing that ulcerative colitis patients are at higher risk to develop colon cancer, and therefore, AOM/DSS-induced tumorigenesis is a most relevant model of human colon cancer. Ethanol-induced promotion of AOM/DSS-induced tumorigenesis suggests that ethanol may influence the colonic mucosal inflammation rather than affecting the action of carcinogen. These results indicate that ethanol consumption does not cause inflammation in the colon, but it delays the post colitis recovery from inflammation. This sustained inflammation may play a key role in the promotion of colonic tumorigenesis.

A number of signaling pathways, including TGFβ/Smad signaling [29], have been implicated in the carcinogenesis of colon. A subset of metastatic human colon cancers expresses high levels of TGFβ [30]. TGFβ receptor activation induces phosphorylation of Smad2 and Smad3 that form a hetero multimeric complex with Smad4 leading to activation of subsequent signaling and regulation of targeted gene expression. Recent study has suggested that Smad2/3 phosphorylation is an important event in colitis-associated colorectal cancer and can serve as a biomarker for colitis-associated colorectal cancer [31]. We examined the effect of ethanol feeding on pSmad levels in colon of AOM/DSS treated mice. Results of this study show that pSmad levels were elevated in the colon of AOM/DSS treated mice; pSmad levels were higher both in the non-tumor hypertrophic mucosa and the tumor tissue. Data also show that ethanol feeding further elevates pSmad levels in the hypertrophic mucosal region. The precise role of TGFβ signaling in ethanol effect is unclear. However, it is likely that the TGFβ signaling is directly related to the sustained inflammation in the ethanol fed-AOM/DSS treated mice.

HIF-1α and VEGF are frequently shown to be overexpressed in numerous types of cancers and are known to be important regulators of angiogenesis [32–35]. Evidence suggests that HIF-1α activation regulates VEGF gene expression, indicating a direct interaction between these two tumor markers [33]. Results of our study show that chronic ethanol feeding increases the level of VEGF in the non-tumor hypertrophic colonic mucosa. VEGF was localized predominantly in the lamina propria of hypertrophic colonic mucosa, and connective tissue regions of the tumors. Similarly, HIF-1α was also elevated dramatically by ethanol feeding in the hypertrophic colonic mucosa of AOM/DSS treated mice. HIF-1α was high also in the tumor tissue, which appeared to be reduced by ethanol feeding. However, images at higher magnification indicated that ethanol feeding increased nuclear localization of HIF-1α in the crypts of colonic mucosa. These results indicate that ethanol feeding enhances tumorigenic cell signaling during AOM/DSS-induced colonic tumorigenesis.

Inflammation is a key factor in the development of many cancers, including colorectal cancers [20, 21]. DSS administration is well established to induce colitis [27], and removal of DSS leads to post colitis healing with nearly complete recovery to normalcy in 4–5 days [36]. In the present study, ethanol was fed only during the post colitis recovery periods. We hypothesized that ethanol delays the recovery from colitis and sustains inflammation in the colonic mucosa. To test this hypothesis we examined colons for mucosal infiltration of neutrophils and macrophages at a precancerous stage, that is 2 weeks after the second course of DSS colitis. Analysis of MPO-positive, Gr1-positive and CD68-positive cells by immunofluorescence microscopy showed an absence of neutrophil and macrophage infiltration in the colonic mucosa of mice treated with ethanol feeding or AOM/DSS administration. This observation suggests that ethanol by itself does not cause colonic mucosal inflammation, and that inflammation by DSS colitis in AOM/DSS treated mice is almost completely alleviated during the 2-week recovery period. However, ethanol feeding delays the post colitis recovery, and sustains inflammation even after 2 weeks of recovery. This sustained inflammation may contribute to the mechanism of ethanol-induced promotion of AOM/DSS-induced tumorigenesis in mouse colon.

Inflammatory cytokines such as IL-1α, IL-6 and TNFα are elevated in colon cancer and play important role in the mechanism of tumorigenesis [20, 21]. IL-6 and TNFα are produced by the activated macrophages, a potential mechanism involved in cancer development [21]. TNFα and IL-1α were found to be elevated in MAM-induced colon carcinogenesis [37]. Colonic mucosal inflammation in DSS-induced colitis is associated with an increase in the production of inflammatory cytokines and chemokines. The level of inflammatory cytokines and chemokines subside during post colitis healing period [36]. We examined the colonic mucosa for the levels of mRNA for IL-1α, IL-6 and TNFα. Ethanol feeding by itself produced no significant effect on mRNA for IL-1α, IL-6 and TNFα. Similarly, cytokine mRNA was unaltered in colonic mucosa of AOM/DSS treated mice, which is consistent with the lack of inflammatory cells in the colons of these mice. Similar to its effect on neutrophil and macrophage infiltration, ethanol feeding increased mRNA levels for cytokines in the colonic mucosa of AOM/DSS treated mice by 4–5 folds. Lack of elevated IL-6 and TNFα in the colon of AOM/DSS treated mice indicates that the mucosa is almost completely recovered from colitis during 2-week recovery period. Elevated expression of cytokines in ethanol fed-AOM/DSS treated mouse colon demonstrates that chronic ethanol feeding delays the healing process and sustains inflammation.

Unlike in colon, in the ileum, ethanol feeding significantly elevated IL-1α, IL-6 and TNFα mRNA levels. This is an interesting observation, suggesting that ethanol does induce inflammatory reactions in the ileum, but not in colon. The mechanism and significance of this observation is unclear at this time as no tumors were detected in the ileum under our experimental conditions. AOM/DSS treatment also elevated the IL-1α, IL-6 and TNFα mRNA in the ileum, indicting that sustained inflammation exists in the ileum of AOM/DSS treated mice. In the ileum, the effect of ethanol on cytokine expression in AOM/DSS treated mice was variable. Ethanol enhanced TNFα expression, whereas it decreased IL-1α expression; IL-6 expression was unaffected by ethanol.

Chemokines and chemokine receptors are known to play a role in the mechanism of cancer metastasis [38]. While chemokines are known to play a key role in the intestinal mucosal homeostasis and pathogenesis of inflammatory bowel diseases [39], chemokines such as CXCL9/MIG and CXCL10/IP-10 are overexpressed in colon cancer [40]. CCL5/RANTES, which is chemotactic for macrophage and T cells, is highly expressed in both human and mouse colon cancers, and contributes to the inflammation and malignant progression [41]. Evidence indicates that CCL5/RANTES helps in immune escape of colorectal cancers. Results of our present study show that ethanol feeding and AOM/DSS treatment significantly elevated CCL5/RANTES mRNA in colon, suggesting that both ethanol and AOM/DSS treatment increase the expression of CCL5/RANTES in colonic mucosa. A combined treatment with ethanol and AOM/DSS induced a synergistic increase in CCL5/RANTES expression, suggesting that CCL5/RANTES may play a role in the mechanism of ethanol-mediated macrophage infiltration and sustained inflammation in AOM/DSS treated mouse colon. The levels of mRNA for CXCL9/MIG and CXCL10/IP-10 were unaffected by either ethanol or AOM/DSS, but they were synergistically elevated by a combination of ethanol feeding and AOM/DSS treatment. CXCL9/MIG and CXCL10/IP-10 are anti-angiogenic chemokines and are likely to suppress tumorigenesis [40]. The significance of ethanol-induced elevation of CXCL9/MIG and CXCL10/IP-10 gene expression is unclear at this time. However, it is likely that these are expressed as a defense against inflammation.

Dramatic reduction in the levels of mRNA for occludin, ZO-1 and Cldn3 in the colon of AOM/DSS treated mice indicates a decline in the expression of tight junction proteins. Immunofluorescence microscopy confirms the depletion of occludin and ZO-1 in the colon by AOM/DSS treatment. Down regulation of cell-cell adhesion proteins is an important event in the pathogenesis of colon cancer. Interestingly, ethanol feeding worsened the effect of AOM/DSS treatment on the expression of tight junction proteins. Increase in the level of Ki-67 in colonic epithelial cells by AOM/DSS treatment and its further enhancement by ethanol feeding suggests that ethanol may promote AOM/DSS-induced increase in proliferative activity of colonic epithelial cells.

## Conclusions

In summary, the results of this study show that chronic ethanol feeding delays post colitis mucosal recovery leading to sustained mucosal inflammation and elevated cytokine and chemokine expression. Sustained inflammation and elevated cytokines and chemokines might play a role in the mechanism of alcohol-mediated promotion of colonic tumorigenesis. Overall, outcome of this study suggests that alcohol consumption by patients with ulcerative colitis may delay the healing and/or recurrence of the disease symptoms. Furthermore, chronic alcohol consumption by patients with the history of ulcerative colitis may elevate the risk of developing colon cancer.

## Abbreviations

AOM: azoxymethane
CCL5/RANTES: chemokine ligand 5
Cldn3: claudin 3
CXCL: C-X-C chemokine ligand
DSS: dextran sulfate sodium
DMH: dimethylhydrazine
EF: ethanol fed
EtOH: ethanol
HIF1α: hypoxia-inducible factor-1α
IL-1: interleukin 1
IL-6: interleukin 6
Ki-67: an antigen marker of cell proliferation
MAM: methylazoxymethanol
MPO: myeloperoxidase
PF: pair fed
SDS: sodium dodecyl sulfate
TNF: tumor necrosis factor
TGF: transforming growth factor
VEGF: vascular endothelial growth factor
ZO-1: zonula occludens 1
